# Supplementation of docosahexaenoic acid (DHA), vitamin D_3_ and uridine in combination with six weeks of cognitive and motor training in prepubescent children: a pilot study

**DOI:** 10.1186/s40795-017-0155-1

**Published:** 2017-04-17

**Authors:** Solvejg L. Hansen, Anina Ritterband-Rosenbaum, Camilla B. Voigt, Lars I. Hellgren, Ann-Dorit M. Sørensen, Charlotte Jacobsen, Line Z. Greve, Katrine D. Jørgensen, Peder E. Bilde, Bente Kiens, Jens B. Nielsen

**Affiliations:** 1 Department of Nutrition, Exercise and Sports, Section of Molecular Physiology, August Krogh Building University of Copenhagen, Universitetsparken 13, 2100 Copenhagen Ø, Denmark; 2 Center for Neuroscience, Panum Institute, University of Copenhagen, Blegdamsvej 3, 2200 Copenhagen N, Denmark; 3 Center for Biological Sequence Analysis, Department of Systems Biology, Technical University of Denmark, Søltofts Plads, 2800 Kgs. Lyngby, Denmark; 4 National Food Institute, Division of Industrial Food Research, Technical University of Denmark, Søltofts Plads, 2800 Kgs. Lyngby, Denmark; 5 The Elsass Institute, Holmegårdsvej 28, 2920 Charlottelund, Denmark

**Keywords:** Nutritional supplement, Training, Children, Blood samples, DHA, Vitamin D, Uridine, Pilot study

## Abstract

**Background:**

Learning and memory have been shown to be influenced by combination of dietary supplements and exercise in animal models, but there is little available evidence from human subjects. The aim of this pilot study was to investigate the effect of combining a motor- and cognitive exercise program with dietary supplementation consisting of 500 mg docosahexaenoic acid (DHA), 10 μg vitamin D_3_ and 1000 mg uridine (DDU-supplement) in 16 prepubescent children (age 8–11 years).

**Methods:**

We designed a randomized, placebo-controlled, double-blinded study lasting 6 weeks in which DDU-supplement or placebo was ingested daily. During the intervention period, all children trained approximately 30 min 3 days/week using an internet-based cognitive and motor training program (Mitii). Prior to and post the intervention period dietary record, blood sampling, physical exercise tests and motor and cognitive tests were performed.

**Results:**

Fourteen of the 16 children completed the intervention and ingested the supplement as required. 6 weeks DDU-supplementation resulted in a significant increase in the blood concentration of vitamin D_2+3_ and DHA (*p* = 0.023 and *p* < 0.001, respectively). Power calculation based on one of the cognitive tasks revealed a proper sample size of 26 children.

**Conclusion:**

All children showed improved performance in the trained motor- and cognitive tasks, but it was not possible to demonstrate any significant effects on the cognitive tests from the dietary supplementation. However, DDU-supplementation did result in increased blood concentration of DHA and vitamin D_2+3_.

**Trial registration:**

Clinical registration ID: NCT02426554 (clinical Trial.gov). January 2015 retrospectively registered.

## Background

Learning and memory require structural changes in neurons and neuronal networks in the form of down- or up-regulation of receptors, membrane channels, neurotransmitters and synaptic connections [1]. Such plastic changes depend on the availability of proteins, fatty acids and carbohydrates involved in the growth of new connections between the neurons. The influence of different dietary supplements on brain function in animals and humans has therefore been the subject of intense scientific interest in the past decade (reviewed in [2]). Most promising among these dietary supplements are substrates for synthesis of the neuronal membrane phospholipids uridine and docosahexaenoic acid (DHA), which have been shown in rodents, independently or in combination, to facilitate development of new synapses and to improve learning and memory abilities [2–6]. DHA supplementation in rodents has also been shown to increase the production of brain-derived neurotrophic factor (BDNF) [7], a mediator of neurogenesis in hippocampus and critical in memory formation [8]. Vitamin D receptors are found in human brain and vitamin D is like DHA able to cross the blood brain barrier (reviewed in [9]). In a systematic review, low vitamin D levels have been associated with decline in cognitive function and higher frequency of dementia [10]. Taken together, research results suggest that DHA, vitamin D and uridine are of importance for optimal brain function.

To our knowledge, no studies have investigated the effect of a combination of vitamin D and DHA supplementation on cognitive abilities in healthy prepubescent children. Some studies have examined the effect of DHA alone in randomized control trials [11–16]. Thus, the available evidence does not present a clear and coherent picture. Three studies have shown a positive effect of DHA supplementation on some cognitive outcomes [15–17] one study has shown mixed results [14] while others report no effect [11–13]. In a recent meta-analysis, the effect of n-3 polyunsaturated fatty acid was evaluated on cognitive outcome and it was concluded that the supplementation may significantly improve cognitive development in infants, but does not improve cognitive performance in children, adults, or in the elderly [18].

Interestingly, DHA supplementation also seems to have synergistic effects together with exercise/physical activity on learning and memory abilities [2, 19, 20]. In addition, a recent analysis of 59 studies in children indicated a significant and positive effect on cognitive outcomes in response to exercise and physical activity alone [21]. However, studies of the effects of a dietary supplement and physical activity like motor- and cognitive exercises in combination in healthy children are lacking.

The aim of the present pilot study was to provide preliminary data of the effect on cognitive and motor abilities by an intervention consisting of repeated motor- and cognitive exercises and daily supplementation of DHA, vitamin D_3_ and uridine (DDU-supplement) in a group of prepubescent children to enable a power calculation. The purpose of the study was further to address; 1) the feasibility of including prepubescent children to participate in repeated bouts of motor- and cognitive exercises combined with extensive testing, and 2) whether a relative small supplementation of vitamin D and DHA can be detected as an increase in blood levels after just 6 weeks of supplementation.

## Methods

### Study site

The study was conducted during winter and spring 2011. Children in 3rd and 4th grade (age 8 to 11 years.) were recruited and written informed consent was obtained from all children and their parents. This study was conducted in accordance with the Declaration of Helsinki and all procedures were approved by The National Committee of Health Research Ethics - the Ethics committee of the Greater Copenhagen area (H-2-2010-061).

### Subjects

Sixteen school children were recruited for the study and met the inclusion criteria, which were age of 8 to 11 year. and no history of neurological or psychiatric disorders. The recruitment period lasted 4 months. All subjects were right-handed according to the Edinburgh Handedness Inventory [22].

Completion rate was 88%. Two children dropped out during the experiment, one due to lack of time and one because he disliked the supplement. Accordingly, 14 children (6 girls and 8 boys) completed the study. Results are from the 14 children unless otherwise stated. Table 1 summarizes the characteristics of the children divided in DDU-group (6 children, 3 girls/3 boys) and placebo group (8 children, 3 girls/5 boys).

**Table 1:** Characteristics

	DDU supplement	Placebo supplement
	Mean + SEM	Mean + SEM
Age (years)	9.5 ± 0.22	9.4 ± 0.18
Weight (kg)	34.58 ± 2.63	33.58 ± 1.53
Height (m)	1.44 ± 0.02	1.42 ± 0.02
BMI (kg/m^2^)	16.66 ± 1.0	16.7 ± 0.56
^a^VO_2_max (l/min)	1.73 ± 0.17	1.86 ± 0.08
^a^VO_2_max (l/min/kg)	0.05 ± 0.002	0.06 ± 0.004

Characteristics for the two intervention groups DDU (6 children) and Placebo (8 children)

^a^VO_2_max: maximal oxygen uptake

### Random assignment of supplement and blinding

All children received a personal code, which was used in the randomization process and in the testing results. All supplement containers (DDU and placebo) were labelled with the codes. The supplier of the supplement held the codes until data analyses were completed. None of the codes were broken before then. Thus, children, parents, investigators and staff were blinded throughout the intervention period.

### Research design

**Fig. 1:**
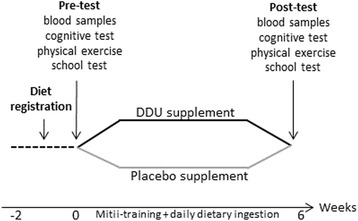
Study design. The children completed dietary registration during four representative days two weeks prior to the pre-test. During the intervention two children dropped out of the study, which resulted in 6 children in the DDU group and 8 children in the Placebo group

A combination of DHA, vitamin D_3_ and uridine (DDU-supplement) or a placebo supplement, containing medium-chain triacylglycerol, was ingested daily in a 6-week period. Children were randomly allocated to start with DDU-supplement or placebo supplement. The supplements were administered at home. The intervention was combined with a cognitive- and motor exercise program, consisting of a number of progressively challenging cognitive- and motor training for 30 min a day, 3 days a week. Prior to the intervention period, four representative days of habitual diet recording were completed. Furthermore, the children filled a food frequency and physical activity questionnaires (FFQ), which covered periods prior to, during and post intervention. Before and after the intervention period, blood sampling and testing of cognitive parameters such as attention, learning and memory were performed. Furthermore, maximal oxygen uptake (VO_2_max) was measured on a treadmill to determine the physical fitness level of the children. The children were told not to change their daily physical activity habits during the study. This was controlled by the FFQs. See Fig. 1.

### Dietary record

Four days of diet recording were completed prior to the intervention period to examine the daily habitual diet composition and energy intake of the children. Electronic scales with one gram of accuracy (OBH NORDICA, attraction, Kitchen scale) and specified registration schedules were used for weighing all intakes. Information and instructions were provided verbally and in writing to the children and a family member by a researcher with several years of training in obtaining dietary records. Representative days for diet recording were planned together with each child and family member. The computer program DANKOST 3000 was used for data analysis of the dietary records. This provided details about energy intake and macro- and micronutrients.

The children and their parents filled out a non-validated FFQ before, during and after the intervention. The FFQ were designed to detect any changes in physical activity level (type, duration and intensity of daily activities) and intake of vitamin D and DHA rich food sources (for example, how often and how much the subject had ingested tuna fish the last 3 days).

### Blood sampling

Blood samples were collected by assistants at the Department of Biochemistry, Copenhagen University Hospital (Rigshospitalet). The children arrived fasting (12 h) at the school laboratory in the morning. The children were allowed to rest for 15 min in sitting position before blood was drawn from an antecubital vein. Plasma or serum was frozen at minus 20 or 80° Celsius for further analysis.

### Testing

#### Math test and reading/comprehension test

All children completed school tests, which included a reading/comprehension task as well as a math test. All exercises were specifically chosen in accordance to the level of 3^rd^ and 4^th^ grade school children. Accuracy and reaction time of each task were noted. The reading/comprehension test is a standardized school test, where children have a maximum of 15 min to complete the task. In order to note the speed of their answers, the colour of the pencil was changed every fifth minute. For both the math test and the reading/comprehension test, the number of errors was assessed. The tests were administered by a researcher, who had several years of experience in performing the tests.

#### Cognitive tests

A psychologist tested the children in different psychological tests specifically chosen to provide outcome measures related to visual learning, memory, attention and executive functions. We used the CogState [23] program, which is a computer based testing apparatus. The program presents tasks with similar levels of difficulty in different ways for pre and post testing, and is therefore well suited for repeated administration. The tests lasted around 30 min in total. The main outcomes from the test were given by reaction time and accuracy.

Attention and working memory were assessed by the One Back and Two Back Tasks. In these tests the children had to recall if a given card was similar to the last or second to last card, respectively.

Visual learning and memory were assessed by a Continuous Paired Associative Learning (CPAL) task in which the children had to memorize positions of up to seven abstract figures on the computer screen.

Executive function, spatial memory and working memory were assessed by a Groton Maze Learning (GML) test. The children had to remember a specific path in a maze, which consisted of 10 x 10 squares. This test was also used as a recall-test after completing the other tests in the CogState program.

Visual attention was also evaluated by an Identification task, in which the children had to give the colour of a card presented on the computer screen as quickly as possible (red card vs. black card).

#### Measurement of maximal oxygen uptake

Physical fitness level was evaluated by a running test on a treadmill before and after the intervention. The children were familiarized with the equipment and test procedure before the running test. During all tests, the children breathed through paediatric masks, adapted to their faces. All children were secured with a safety belt (Teddy pants, Liko, Sweden) during the entire test. Heart rate (HR) was continuously recorded (polar Electro, Finland). Respiratory gas exchanges were measured breath-by-breath using an automatic gas-analysis system (CPX MedGraphics, USA) to determine oxygen uptake (VO_2_) and respiratory exchange ratio (RER). Calibration of the O_2_ and CO_2_ analysis systems was performed before testing using ambient air and a mix of known O_2_ and CO_2_ concentrations (15% O_2_ and 5.8% CO_2_). The tube flowmeter was calibrated using a 3 L syringe. For data analysis values were recorded every 5 s.

The children performed an incremental running test to exhaustion on the treadmill. The protocol started with 1 min running at an individual maximal speed (9–11.5 km/h) on slope 0% followed by stepwise 1% incline every 1 min until exhaustion. Criteria for reaching exhaustion were: RER > 1.00, a plateau in VO_2_ despite increasing slope and unable to continue running despite verbal encouragement. Both RER and VO_2_ could be evaluated throughout the test due to the use of online measurements. The test was overseen by a human physiologist.

### DDU-supplement and placebo supplement

DDU-supplement consisted of a 10 ml oil-in-water (o/w) emulsion of 500 mg DHA in triacylglyceride form, 10 μg vitamin D_3_, 1000 mg uridine and 0.5 g blue berry extract. We chose the dose of DHA based on a recommendation of a daily intake of 500 mg polyunsaturated fatty acids (DHA + EPA) in healthy subjects by the International Society for Study of Fatty Acids and Lipids (ISSAFL) [24]). The Nordic recommendation of daily vitamin D intake is 10 μg. Since there is no known recommended level for Uridine, we chose a dose well below that used in animal research. Holguin et al. 2008 used a dose corresponding to 0.03% of body weight and we consequently decided to use 1 g of uridine, which corresponds to 0.003% of body weight [25]. Blueberry extract was added to protect DHA against oxidation and at the same time to provide a berry like colour of the emulsion. Placebo consisted of a 10 ml o/w emulsion of 2 g medium-chain triglycerides (MCT) oil and artificial colouring. Whey protein was used as emulsifier in both types of emulsions. In addition, both emulsions contained synthetic blueberry flavour to make the flavour and odour of the two emulsions similar. An overview of the different ingredients and their concentrations in the two different emulsions is shown in Table 2. Prior to administering the supplements to the children, a group of naive adults tasted the two emulsions to ensure that they looked and tasted alike.

**Table 2:** Composition of supplements shown in WT % (weight solute/weight total)

	DDU supplement	Placebo supplement
Hydrophilic ingredients		
Water	62.1	58.9
Whey protein	1.01	1.01
Blueberry flavour	5.00	
Uridine	10.0	
Artificial flavour	1.69	0.85
Red colour		13.1
Blue colour		1.05
Green colour		4.18
Lipophilic ingredients		
High DHA oil^a,b^	17.3	
Vitamin D3^a^	2.90	
MCT oil		20.1

Composition of DDU-supplement and Placebo emulsions. DHA, Docosahexaenoic acid. MCT, medium-chain triacylglycerol

^a^The amount of these ingredients was adjusted according to their purity to give the desired amount of bioactive compounds (DHA: 0.5 g; Vitamin D3: 10 μg) in 10 ml of the DDU emulsion

^b^Incromega DHA 500TG

Production: DDU-supplement and placebo emulsions were produced in 2-steps: pre-emulsification and homogenization. First, whey protein was solubilized in the water and other hydrophilic ingredients were thereafter added to the whey protein–water solution (DDU-supplement: uridine and blueberry extract, artificial flavour; Placebo: artificial colour and flavour). For pre-emulsification, the aqueous solutions were stirred with an Ultra-Turrax (Janke & Kunkel IKA-Labortechnik, Staufen, Germany) and the oil mixture (DDU-supplement: DHA 500TG and vitamin D_3_) or oil (Placebo: MCT oil) was added during the first min of the 2 min total mixing. Pre-emulsions were then homogenized using a two-valve table homogenizer at a pressure of 225 bar (GEA Niro Soavi Spa, Parma, Italy). Produced emulsions were bottled (10 mL), purged with nitrogen to limit lipid oxidation, sealed and pasteurized in a water bath (72 °C). All bottles were cooled at 5 °C and thereafter stored at −20 °C. The children received the DDU-supplement or placebo supplement for three weeks at a time and stored them in their private freezer (−18 °C) until 24 h before ingestion. During the last 24 h until ingestion the supplements were kept in the refrigerator (5 °C). The supplements were ingested daily in the morning together with at least 100 ml of yoghurt or orange juice as part of their breakfast. The children did not report any side effects of either the DDU-supplement or the placebo supplement. A few of the children noticed taste and texture differences between DDU and placebo, but were not able to identify which supplement corresponded to the active product or placebo.

Materials: Oils used for DDU-supplement (Incromega DHA 500TG, 58% DHA) and Placebo (MCT, medium chain length C_6_-C_12_ triglycerides, where of C_8_ 56% and C_10_ 43%, 99.3% triglyceride) were supplied from CRODA (East Yorkshire, England) and Sasol Germany GmbH (Witten, Germany), respectively. Vitamin D_3_, Uridine and blueberry flavour were purchased from a local dietary shop, Yamasa Corporation (Chiba, Japan) and DENK Ingredients GmbH (Munich, Germany), respectively. Artificial flavor and whey protein were donated by A/S Einar Willumsen (Brøndby, Denmark) and Arla Foods (Viby J, Denmark), respectively. Artificial colors (Dr. Oetker: red, blue and green) were purchased from a local super market.

### Training procedure

The children completed all training sessions at home using a recent developed internet based cognitive- and motor training system (Move It To Improve It: Mitii [26]). The program consisted of a number of progressively challenging cognitive- and motor training modules in which the child used visual information, solved a cognitive problem (i.e. mathematical question, memory related task or similar) and responded with a motor act (i.e. bend to pick up needle and blow up balloon with the right answer). They trained 30 min per day, three days a week. Examples of modules of Memory: the children had to memorize a specific order of images and Mathematics: the children had to solve arithmetic tasks as fast as possible. Further details have been described earlier [26]. All children practiced the same program. Data of the task performance were collected on a server for offline analysis.

## Blood analysis

25(OH)D was used as measurement of plasma vitamin D_2+3_ concentration [27], and was measured by a competitive chemiluminescens immunoassay on a ImmunoDiagnosticSystem (iSYS).

DHA (C22:6, n-3) in plasma was analysed as described earlier [28]. Briefly, plasma lipids were extracted using a modification of the Folch-method, and fatty acid methyl esters (FAME) were produced using a BF_3_-catalyzed method, in which hydroquinone is added as antioxidant [29]. The method has been validated for DHA, and does not induce double-bond losses. The mass-percentage contribution of DHA to the total plasma FAME-pool was analysed using GC-FID, as described earlier [28].

Plasma BDNF concentration was measured by a commercial available kit (Cat. No. CYT306, Chemicon International Chomikine) by ELISA (Millipore, Corporation).

## Power analysis

Power analysis was performed on one of the cognitive tests in order to determine the sample size necessary to obtain a statistically significant difference between the two groups.

The sample size (N) is determined by:

$$ N=2{\left(\sigma \frac{Z_{1- a/\left(2\tau \right)}+{Z}_{1-\beta}}{\mu A-\mu B}\right)}^2 $$

σ = standard deviation

τ = number of pairwise comparison

α = type I error (0.05)

β = type II error (0.80)

μ = mean **Δ** outcome from the groups

## Statistics and calculations

All data were analysed using SigmaPlot (version 11.0, SYSTAT Software, San Jose, CA, USA). Data are expressed as mean ± SEM. Data were evaluated using two-way ANOVA with repeated measures for both intervention (DDU-supplement/placebo) and time (Pre/Post intervention). To test for differences between boys and girls an unpaired t test was performed when variables were independent of time and intervention. A Holm-Sidak test was used as a post hoc test.

The association between vitamin D intake and plasma vitamin D level and relationships between dietary saturated fatty acids, added sugars, and cognitive function were investigated using the Pearson product moment correlation. A significance of *P <* 0.05 was chosen.

## Results

### Subjects

We found that our randomization process was acceptable as the two groups (DDU and placebo) did not differ significantly from each other in their baseline characteristics (average age, body weight, body mass index (BMI) and maximal oxygen uptake (VO2max) (ref. Table 1).

### Habitual diet

**Fig. 2:**
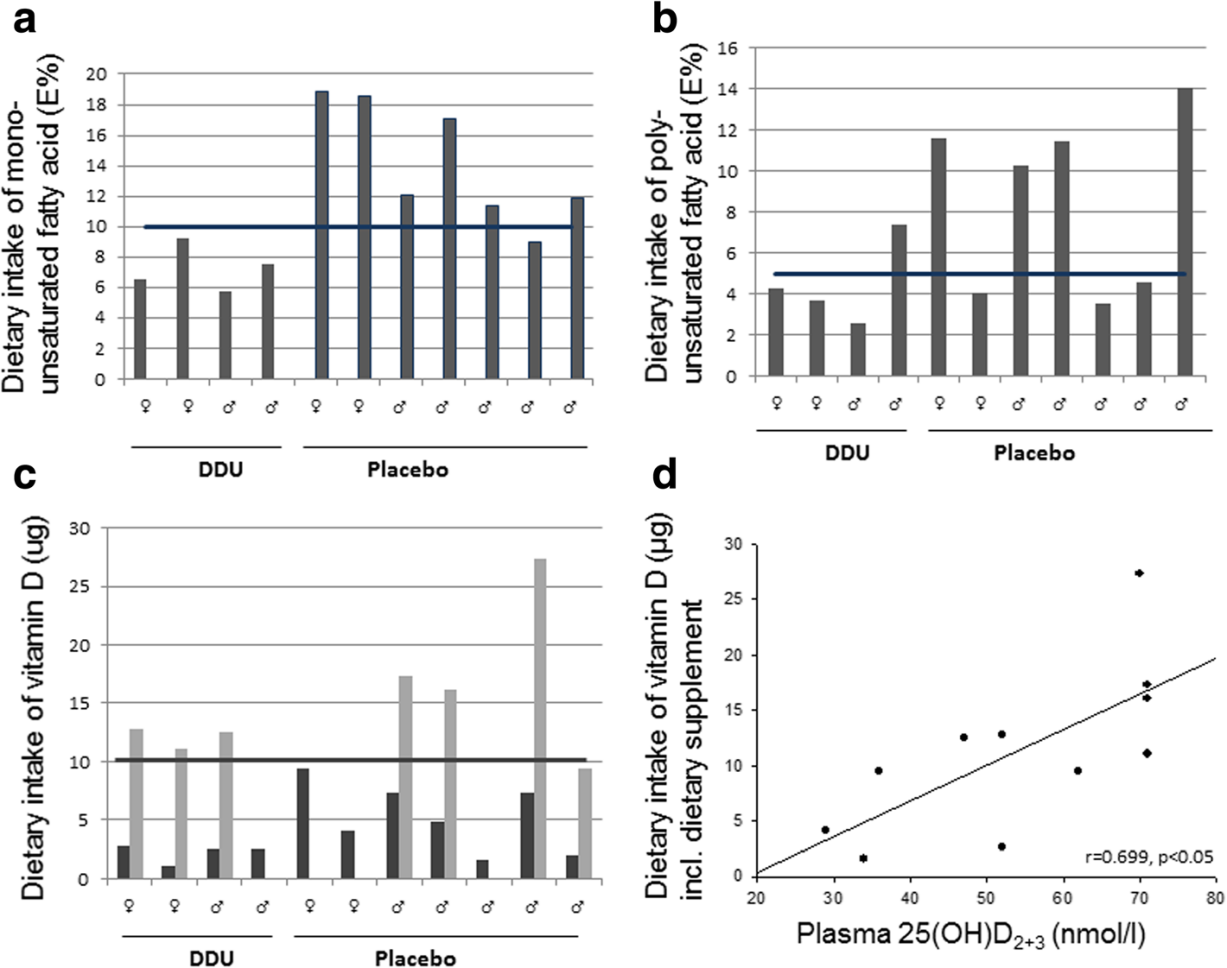
Individual dietary intake of mono- and polyunsaturated fatty acids and vitamin D. 1–4 represents girls and 5–11 represents boys. Bold line represents daily minimum recommended levels of intake monounsaturated fatty acid intake (E%) (**a**), *black horizontal line* indicates recommended level at 10–15 E% [37]. Polyunsaturated fatty acid intake (E%) (**b**), black horizontal line indicates recommended level at 5–10 E% [37]. Vitamin D (μg) intake (**c**) with (*grey bars*) and without (*black bars*) vitamin supplement (7 out of 11), *black horizontal line* indicates recommended daily intake at 10 μg [37]. **d** Correlation between dietary intake of vitamin D incl. dietary supplement and plasma 25(OH)D_2+3_ (vitamin D_2+3_) concentration in blood. Pearson product moment correlation is presented on the graph

All data from the dietary records are presented as the average values from the total group of children and given as average data according to sex (see Table 3 macronutrients and Fig. 2).

**Table 3:** Macronutrients before intervention

	Girls + boys	Girls	Boys	RI^a^
	Mean ± SEM	Mean ± SEM	Mean ± SEM	
Energy intake (KJ)	7797.4 ± 462.1	6733.3 ± 400.9	8405.5 ± 586.3	Girls: 8600 Boys: 9300
Energy intake (kJ/kg bw.)	232.1 ± 17.8	208.0 ± 17.2	245.8 ± 25.7	
Macronutrients				
Protein (E%)	16.0 ± 0.5	17.2 ± 0.2	15.3 ± 0.7	10-20
Fat (E%)	27.5 ± 1.3	30.7 ± 1.9	25.6 ± 1.4	25-40
Carbohydrate (E%)	56.6 ± 1.5	52.1 ± 2.0	*59.1 ± 1.2	
Lipid profile				
Saturated fatty acid (E%)	8.3 ± 0.6	9.0 ± 0.7	7.9 ± 0.8	<10
Monounsaturated fatty acid (E%)	11.6 ± 1.4	13.3 ± 3.2	10.7 ± 1.4	10 → 20
Polyunsaturated fatty acid (E%)	7.0 ± 1.2	8.4 ± 2.6	6.3 ± 1.3	5 → 10
Cholesterol (mg)	217.7 ± 35.3	266.4 ± 52.1	189.8 ± 46.2	<300
Added sugar (E%)	3.48 ± 0.66	3.80 ± 1.84	3.30 ± 0.37	<10
Fibers (g)	26.0 ± 2.1	21.1 ± 1.6	28.8 ± 2.7	2-3 g/Mj

Macronutrients given by an average of four independent days of 24 h dietary weighing for girls and boys together (*n* = 11) and girls (*n* = 4) and boys (*n* = 7) separately

*p < 0.05, vs. girls (t test), bw = body weight. E%, percentages of total energy intake

^a^RI: Recommended intake for a child aged 10–11 years [37]

Diet records from three children were excluded because of insufficient registration (less than two days of recording). Eight of the children supplemented their daily diet with vitamins and minerals. All children were well below the upper limit for recommended intake of vitamin D regardless of extra supplementation. The dietary records are reported without these individual supplements when nothing else is noted. There was no cross-sectional relationship (at pretest) between dietary saturated fatty acids, added sugars, and cognitive function (*p* > 0.05).

Energy intake was 208.0 ± 17.2 kJ/kg body weight (bw) in girls and 245.8 ± 25.7 kJ/kg bw in boys (not statistically different; *p* = 0.3). The composition of the habitual diet averaged 56.6 ± 1.5 energy percentage (E%) of carbohydrates, 27.5 ± 1.3 E% fat and 16.0 ± 0.5 E% protein. The boys consumed a higher E% of carbohydrates (*p* < 0.05) and a lower E% fat (*p* = 0.056) than the girls (Table 3).

The intake of fatty acids in the diet was low in relation to recommendations. Despite of this, the average intake of mono- and polyunsaturated fatty acids was within the range of recommended levels. 45% of the children were below recommended intake of both mono – and polyunsaturated fatty acids (Fig. 2a and b).

The average daily vitamin D intake was below the recommended level for both genders (mean 4.15 ± 0.84 μg, recommended level; 10 μg). Seven children supplemented their daily diet with vitamins and minerals and thereby reached the recommended level of vitamin D intake (Fig. 2c). Evaluation of the FFQs showed that none of the children changed their diet habits related to food items containing vitamin D or DHA during the study period.

### Blood parameters

**Fig. 3:**
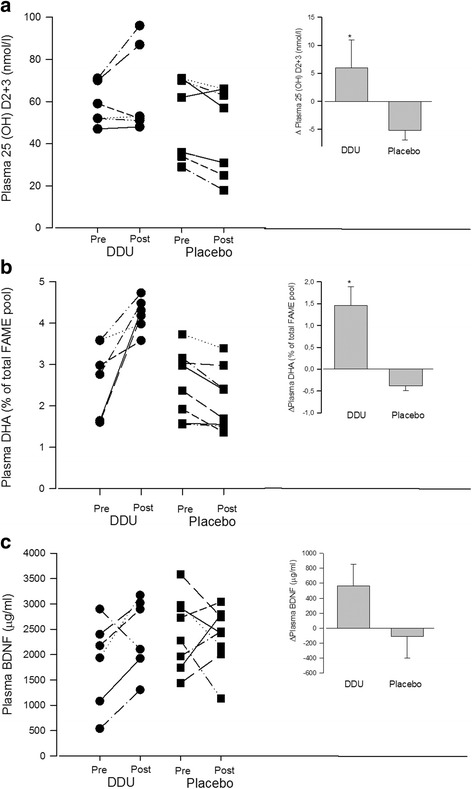
Plasma concentrations of vitamin D2 + 3, docosahexaenoic acid (DHA) and brain-derived neurotrophic factor (BDNF). Vitamin D_2+3_ plasma concentration (**a**) DHA plasma content (**b**), and BDNF plasma concentration (**c**) before and after DDU- and placebo supplement. Right upper corner: Delta concentrations of vitamin D_2+3_ (**a**), DHA (**b**) and BDNF (**c**) in relation to interventions. Data are mean ± SEM

Before the DDU-supplementation the average plasma concentration of vitamin D_2+3_ (25(OH)D) was 58.5 ± 4.1 nmol/l. It increased to 64.5 ± 8.6 nmol/l (*p* = 0.023) after DDU-supplementation, but remained unchanged in the placebo trial (pre: 51.13 ± 6.7 nmol/l, post: 44.6 ± 7.2 nmol/l) (Fig. 3a).

Before the DDU-supplementation trial the average plasma DHA concentration was 2.69 ± 0.36% of total FAME-pool and increased to 4.15 ± 0.21% of total FAME-pool (*p* < 0.001). In the placebo trial plasma DHA concentration was unchanged (pre: 2.54 ± 0.28, post: 2.16 ± 0.27% of total FAME-pool). Delta plasma concentration of DHA was significantly larger in DDU-supplementation trial compared to placebo trial (Fig. 3b).

BDNF concentration remained unchanged following both the DDU- and the placebo supplement (Fig. 3c).

A correlation was found between dietary vitamin D intake and plasma vitamin D_2+3_ concentration (Fig. 2d, *r* = 0.7, *P* < 0.05).

## Training intervention

Compliance to Mitii training was good. Out of 18 possible training sessions the children completed on average 16 sessions during the intervention period.

**Fig. 4:**
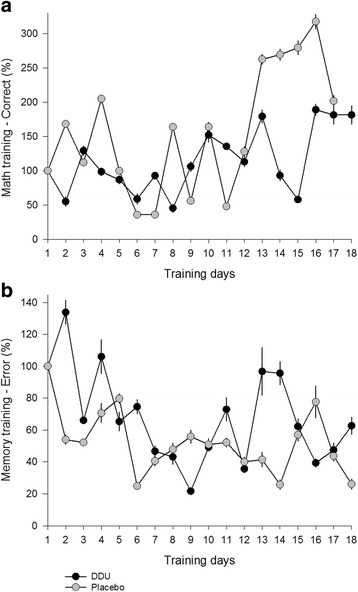
Performance in two Mitii modules. Mathematics (**a**) and Memory (**b**) during the intervention periods when children received DDU-supplement (closed circles) or placebo (open circles). The mathematic task is given by an average increase in percentage of correct responses each possible training day (note: nobody from the placebo group trained day 18 therefore no results are provided). The memory task is given by an average decrease in percentage of errors during each possible training day. The error bars indicate SEM for each group

The training program resulted in significant improvement in performance of motor-and cognitive tasks with no difference in relation to dietary supplementation. Examples of progression in the performance of two of the training modules are shown in Fig. 4.

### School test

Reaction time for completing reading/comprehension test was significantly reduced following the motor and cognitive exercise intervention (*p* = 0.008), with no decline in accuracy. Reaction time of the math test was improved (*p* = 0.016) following DDU-supplement, but not in response to placebo. There was no decline in accuracy following the interventions.

### Cognitive tests

**Fig. 5:**
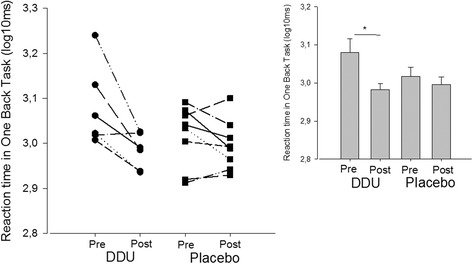
Performance in a Cogstate task. Reaction time before and after DDU- and placebo supplement in the One Back Task. A lower reaction time indicates better performance. Individual scores (left side of the figure) and mean score values (right upper corner) are displayed. The SEM is given

There was in general effect of improvement in performance in the cognitive tasks following both DDU and placebo intake, suggesting that this was a simple test-retest effect. Only one of the subtests showed a significant change following the intervention (*p* < 0.05) (Fig. 5).

### Maximal oxygen uptake and physical activity level

Physical fitness status was evaluated by the measurement of maximal oxygen uptake and supported by evaluation of physical activity level from the FFQ.

Maximal oxygen uptake remained unchanged during the intervention both in the DDU-supplementation group (pre; 1.73 ± 0.17 l/min, post: 1.865 ± 0.08 l/min) and the placebo group (placebo pre: 1.86 ± 0.21 l/min, post: 1.98 ± 0.23 l/min).

Analyses of FFQ revealed no changes in habitual daily activity level during the intervention period.

## Power analysis

Data from one of the cognitive tasks (One Back Task) were used to calculate the sample size necessary to determine significant differences between the two groups (DDU and Placebo):

$$ \mathrm{N}=2\left(3.64\frac{Z_{1-0.05/2}+{Z}_{1-0.8}}{4.62\hbox{-} 1.74}\right) $$

This revealed that a sample size of 26 children would be necessary to determine significant differences between the DDU and placebo groups given the variability and effect size in the test.

## Discussion

The main purpose of the study was to provide preliminary data on the possible effect of DDU supplementation in combination with cognitive-motor training on the cognitive and motor abilities of prepubescent children. This enabled us to determine the feasibility of a randomized clinical study and perform a power analysis.

Our pilot study does support the possibility of implementing a quite comprehensive protocol with dietary questionnaires combined with physical and cognitive testing, including blood sampling, prior to and post diet supplementation and cognitive motor training intervention.

The children generally showed a high compliance to the study with only two children dropping out for reasons that had little to do with the study protocol. All children and their families were willing to supplement their daily food intake with the DDU or placebo supplement. We believe that a similar compliance is to be expected also in a larger study with more children. We were in contact with the families on a weekly or biweekly basis, but this was primarily by mail or phone, which can easily be implemented in a larger study.

Since we have data from only 14 children, all conclusions should be made with caution. Our power calculation indicated that a sample size of at least 26 children would have been necessary to detect true significant effects with the tests that we performed here. With a 10-15% drop-out ratio (2 out of 16) a somewhat larger sample size probably should be aimed for.

Only few studies have investigated the effect of DHA supplementation on cognitive abilities in healthy children and the results are conflicting [11–17]. The NEMO study [11] included a total of 644 healthy children from Australia and Indonesia aged 6–10 years. One group of children (n = 165) was provided with a supplement of vitamin: A, B-6, B-12, C, folate zinc and iron, DHA (88 mg/d) and eicosapentaenoic acid (EPA, 22 mg/d) during a period of 12 months. Higher plasma micronutrient levels and plasma DHA concentrations were found and the children improved verbal learning and memory after the dietary supplementation [11]. These findings are consistent with the double-blinded RCT by Portillo-Reyes et al. (2014), who investigated cognitive outcomes in 50 children aged 8–12 years. Half of the group received 60 mg DHA and 90 mg EPA and the other half received placebo. Significant changes between the two groups were observed in visual-perceptive capacity, attention and executive functions following three month of intervention [17]. In contrast, a double-blinded RCT by Kennedy et al. [12] in 88 healthy children aged 10–12 years. showed no effect on a number of cognitive skills after 8 weeks intake of supplement, containing two different daily doses of DHA (400 mg, 1000 mg) compared to placebo. In that study no dietary recording or blood sampling was performed [12]. None of these studies accounted for changes in daily physical activity level, although exercise has been shown to have significant, positive effects on cognitive function [21]. McNamara et al. (2010) found altered activity in cortical attention networks in a group of children (aged 8–10 year.) following 8 weeks of either a low (400 mg pr. day) or high (1200 mg pr. day) dose of DHA compared to placebo. However, all children displayed the same level of performance in a cognitive task regardless of whether they received DHA or placebo. One important finding from the study by McNamara et al. (2010) was that blood levels of DHA increased significantly after only 8 weeks of DHA ingestion [16]. In the present study a significant increase in plasma DHA (>50%) was observed in response to only 6 weeks of DDU-supplementation. Similar to the study by McNamara (2010), this was not associated with significant improvements in cognitive skills when compared to placebo (besides one test) [16]. It is likely that a longer intervention period is necessary to demonstrate significant cognitive changes as also suggested by Stonehouse (2014) who recommended a minimum of 16 weeks [30].

DHA may facilitate improvements in cognitive skills by increasing BDNF production in the brain. BDNF is believed to be an important mediator of neurogenesis in hippocampus and critical in memory formation [8]. DHA mediated BDNF production has been reported in rodents [7]. It is known that BDNF crosses the blood barrier in both directions. Thus, plasma/serum drawn from an antecubital vein is believed to reflect the BDNF level in the brain [31]. We found no effect of supplementation on plasma BDNF concentration (Fig. 2c), despite an increase in plasma DHA concentration (Fig. 2b). Aerobic exercise and resistance training have been shown to increase serum BDNF concentration in adults in some studies [32, 33] but not in all [34]. The habitual physical fitness level of the children in the present study, as indicated by their maximal oxygen uptake, remained unchanged during the intervention period. Thus, the training may not have been sufficiently intensive to facilitate BDNF production. This may have contributed to the lack of a combined effect of the supplement and the training on cognitive performance.

The baseline plasma concentration of vitamin D_2+3_ was 58.5 ± 4.1 nmol/l (23.4 ± 1.6 ng/ml) in the DDU group and 51.1 ± 6.7 nmol/l (20.5 ± 2.7 ng/ml) in the placebo group. This is considered as vitamin D insufficiency [35]. 90-95% of vitamin D plasma concentrations are caused by exposure to sunlight [27]. The study was initiated in wintertime and stopped during the spring. During this period, only little synthesis of vitamin D occurs in the skin of people living at the latitude of Denmark (54-58°N) [36]. At the same time adequate intake of Vitamin D may be a challenge due to limited availability of food sources containing vitamin D [27]. This is a likely reason for the low vitamin D concentration found in the children in the present study and points to the importance of supplementing the daily diet with Vitamin D during the winter in countries at high latitudes. Our data indicated that supplementation of 10 μg vitamin D_3_ per day in 6 weeks increased plasma levels of vitamin D2 + 3 to 64.5 ± 8.6 nmol/l, which corresponds to 25.8 ± 3.5 ng/ml. This is still below the insufficiency threshold at 29 ng/ml [27]. A higher amount or a longer supplementation period should be considered in future studies.

## Conclusions

We find that it is feasible to combine daily supplementation and cognitive- and motor training during 6 weeks in prepubescent children.

All children showed improved performance in the trained motor- and cognitive tasks, but it was not possible to demonstrate any significant effects on the cognitive tests from the dietary supplementation. However, DDU-supplementation did result in increased blood concentration of DHA and vitamin D_2+3_.

## Abbreviation

BDNF: Brain Derived Neurotrofic Factor
DDU: Vitamin D/DHA and Uridine
DHA: Docosahexaenoic acid
FAME: Fatty acids methyl esters
FFQ: Food Frequency and physical activity Questionnaire
HDL: High density lipoprotein cholesterol
HR: Heart Rate;Respiratory exchange rate
LDL: Low density lipoprotein cholesterol
MCT: Medium chain tryglyceride
Mitii: Move It To Improve It
NEFA: Non-esterified Fatty acids
TG: Triglyceride

## References

[1] Mayford M, Siegelbaum SA, Kandel ER. Synapses and memory storage. Cold Spring Harb Perspect Biol 4. 2012;10.1101/cshperspect.a005751 [doi]. 10.1101/cshperspect.a005751 PMC3367555 22496389

[2] Gomez-Pinilla F (2008). Brain foods: the effects of nutrients on brain function. Nat Rev Neurosci.

[3] Jiang LH, Shi Y, Wang LS, Yang ZR (2009). The influence of orally administered docosahexaenoic acid on cognitive ability in aged mice. J Nutr Biochem.

[4] Sakamoto T, Cansev M, Wurtman RJ (2007). Oral supplementation with docosahexaenoic acid and uridine-5’-monophosphate increases dendritic spine density in adult gerbil hippocampus. Brain Res.

[5] Wurtman RJ, Cansev M, Ulus IH (2009). Synapse formation is enhanced by oral administration of uridine and DHA, the circulating precursors of brain phosphatides. J Nutr Health Aging.

[6] Wurtman RJ, Cansev M, Sakamoto T, Ulus IH (2009). Use of phosphatide precursors to promote synaptogenesis. Annu Rev Nutr.

[7] Wu A, Ying Z, Gomez-Pinilla F (2004). Dietary omega-3 fatty acids normalize BDNF levels, reduce oxidative damage, and counteract learning disability after traumatic brain injury in rats. J Neurotrauma.

[8] Rothman SM, Griffioen KJ, Wan R, Mattson MP (2012). Brain-derived neurotrophic factor as a regulator of systemic and brain energy metabolism and cardiovascular health. Ann N Y Acad Sci.

[9] Harms LR, Burne TH, Eyles DW, McGrath JJ (2011). Vitamin D and the brain. Best Pract Res Clin Endocrinol Metab.

[10] Van der Schaft J, Koek HL, Dijkstra E, Verhaar HJ, van der Schouw YT, Emmelot-Vonk MH (2013). The association between vitamin D and cognition: a systematic review. Ageing Res Rev.

[11] Osendarp SJ, Baghurst KI, Bryan J, Calvaresi E, Hughes D, Hussaini M, Karyadi SJ, van Klinken BJ, van der Knaap HC, Lukito W, Mikarsa W, Transler C, Wilson C (2007). Effect of a 12-months micronutrient intervention on learning and memory in well-nourished and marginally nourished school-aged children: 2 parallel, randomized, placebo-controlled studies in Australia and Indonesia. Am J Clin Nutr.

[12] Kennedy DO, Jackson PA, Elliott JM, Scholey AB, Robertson BC, Greer J, Tiplady B, Buchanan T, Haskell CF (2009). Cognitive and mood effects of 8 weeks’ supplementation with 400 mg or 1000 mg of the omega-3 essential fatty acid docosahexaenoic acid (DHA) in healthy children aged 10–12 years. Nutr Neurosci.

[13] Baumgartner J, Smuts CM, Malan L, Kvalsvig J, van Stuijvenberg ME, Hurrell RF, Zimmermann MB (2012). Effects of iron and n-3 fatty acid supplementation, alone and in combination, on cognition in school children: a randomized, double-blind, placebo-controlled intervention in South Africa. Am J Clin Nutr.

[14] Kirby A, Woodward A, Jackson S, Wang Y, Crawford MA (2010). A double-blind, placebo-controlled study investigating the effects of omega-3 supplementation in children aged 8–10 years from a mainstream school population. Res Dev Disabil.

[15] Richardson AJ, Burton JR, Sewell RP, Spreckelsen TF, Montgomery P (2012). Docosahexaenoic acid for reading, cognition and behavior in children aged 7–9 years: a randomized, controlled trial (the DOLAB Study). PLoS One.

[16] McNamara RK (2010). DHA deficiency and prefrontal cortex neuropathology in recurrent affective disorders. J Nutr.

[17] Portillo-Reyes V, Perez-Garcia M, Loya-Mendez Y, Puente AE (2014). Clinical significance of neuropsychological improvement after supplementation with omega-3 in 8–12 years old malnourished Mexican children: a randomized, double-blind, placebo and treatment clinical trial. Res Dev Disabil.

[18] Jiao J, Li Q, Chu J, Zeng W, Yang M, Zhu S (2014). Effect of n-3 PUFA supplementation on cognitive function throughout the life span from infancy to old age: a systematic review and meta-analysis of randomized controlled trials. Am J Clin Nutr.

[19] Wu A, Ying Z, Gomez-Pinilla F (2008). Docosahexaenoic acid dietary supplementation enhances the effects of exercise on synaptic plasticity and cognition. Neuroscience.

[20] Wu A, Ying Z, Gomez-Pinilla F (2013). Exercise facilitates the action of dietary DHA on functional recovery after brain trauma. Neuroscience.

[21] Fedewa AL, Ahn S (2011). The effects of physical activity and physical fitness on children’s achievement and cognitive outcomes: a meta-analysis. Res Q Exerc Sport.

[22] Oldfield RC (2071). The assessment and analysis of handedness: the Edinburgh inventory. Neuropsychologia.

[23] CogState 2007, CogState Inc, 10th Floor, 195 Church Street, New haven CT 06510, USA

[24] http://www.issfal.org/statements/pufa-recommendations. Online Source. Assessed 14 Feb 2017.

[25] Holguin S, Martinez J, Chow C, Wurtman R (2008). Dietary uridine enhances the improvement in learning and memory produced by administering DHA to gerbils. FASEB J.

[26] Bilde PE, Kliim-Due M, Rasmussen B, Petersen LZ, Petersen TH, Nielsen JB (2011). Individualized, home-based interactive training of cerebral palsy children delivered through the Internet. BMC Neurol.

[27] Holick MF (2005). The vitamin D epidemic and its health consequences. J Nutr.

[28] Drachmann TD, Mathiessen JH, Hellgren LI (2007). The source of dietary fatty acids alters the activity of secretory sphingomyelinase in the rat. Eur J Lipid Sci Technol.

[29] Hamilton JR, Hamilton S (1992). Extraction of lipids and derivative formation, in Lipid Analysis - A Practical Approach.

[30] Stonehouse W (2014). Does consumption of LC omega-3 PUFA enhance cognitive performance in healthy school-aged children and throughout adulthood? Evidence from clinical trials. Nutrients.

[31] Pan W, Banks WA, Fasold MB, Bluth J, Kastin AJ (1998). Transport of brain-derived neurotrophic factor across the blood–brain barrier. Neuropharmacology.

[32] Araya AV, Orellana X, Godoy D, Soto L, Fiedler J (2013). Effect of exercise on circulating levels of brain-derived neurotrophic factor (BDNF) in overweight and obese subjects. Horm Metab Res.

[33] Kuo FC, Lee CH, Hsieh CH, Kuo P, Chen YC, Hung YJ. Lifestyle modification and behavior therapy effectively reduce body weight and increase serum level of brain-derived neurotrophic factor in obese non-diabetic patients with schizophrenia. Psychiatry Res. 2012. doi:10.1016/j.psychres.2012.11.020. 10.1016/j.psychres.2012.11.020 23219101

[34] Swift DL, Johannsen NM, Myers VH, Earnest CP, Smits JA, Blair SN, Church TS (2012). The effect of exercise training modality on serum brain derived neurotrophic factor levels in individuals with type 2 diabetes. PLoS One.

[35] Nimitphong H, Holick MF (2011). Vitamin D, neurocognitive functioning and immunocompetence. Curr Opin Clin Nutr Metab Care.

[36] Brot C, Vestergaard P, Kolthoff N, Gram J, Hermann AP, Sorensen OH (2001). Vitamin D status and its adequacy in healthy Danish perimenopausal women: relationships to dietary intake, sun exposure and serum parathyroid hormone. Br J Nutr.

[37] Nordic council of Ministers. Nordic Nutrition Recommendations 2012, Integrating nutrition and physical activity. 2014.

